# Cost Function Determination for Human Lifting Motion *via* the Bilevel Optimization Technology

**DOI:** 10.3389/fbioe.2022.883633

**Published:** 2022-05-20

**Authors:** Biwei Tang, Yaling Peng, Jing Luo, Yaqian Zhou, Muye Pang, Kui Xiang

**Affiliations:** Intelligent System Research Institute, School of Automation, Wuhan University of Technology, Wuhan, China

**Keywords:** inverse optimization control, bilevel optimization, direct collocation, particle swarm optimization, human lifting motion

## Abstract

Investigating the optimal control strategy involved in human lifting motion can provide meritorious insights on designing and controlling wearable robotic devices to release human low-back pain and fatigue. However, determining the latent cost function regarding this motion remains challenging due to the complexities of the human central nervous system. Recently, it has been discovered that the underlying cost function of a biological motion can be identified from an inverse optimization control (IOC) issue, which can be handled *via* the bilevel optimization technology. Inspired by this discovery, this work is dedicated to studying the underlying cost function of human lifting tasks through the bilevel optimization technology. To this end, a nested bilevel optimization approach is developed by integrating particle swarm optimization (PSO) with the direction collocation (DC) method. The upper level optimizer leverages particle swarm optimization to optimize weighting parameters among different predefined performance criteria in the cost function while minimizing the kinematic error between the experimental data and the result predicted by the lower level optimizer. The lower level optimizer implements the direction collocation method to predict human kinematic and dynamic information based on the human musculoskeletal model inserted into OpenSim. Following after a benchmark study, the developed method is evaluated by experimental tests on different subjects. The experimental results reveal that the proposed method is effective at finding the cost function of human lifting tasks. Thus, the proposed method could be regarded as a paramount alternative in the predictive simulation of human lifting motion.

## 1 Introduction

Lifting motion plays a paramount role in our daily lives and has been found to be one key incentive to pain and fatigue in the human neck and upper limbs (Mombaur et al., 2019). So far, it has been discovered that human lifting behavior complies with an optimal pattern regulated by the central nervous system (CNS) (Chang et al., 2001; Xiang et al., 2021). This discovery implies that an optimal control strategy is more likely involved in human lifting motion. Revealing this potential strategy may not only benefit the development of biomechanics but also provide valuable insights on the bionic control of different wearable robot devices to reduce human physiological discomforts caused by lifting tasks (Mark et al., 2019). However, the complexities of the human CNS and intersubject variances lead investigating the optimal control issue, particularly in terms of establishing the cost function regarding human lifting motion, to be difficult.

Recently, different methods have been devised by numerous researchers to expose the optimal control strategies with respect to different human motions. Among those currently existing methods, predictive musculoskeletal simulation *via* dynamic optimization could be one of the most formidable approaches, thanks to its ability to formulate the dynamic optimization issue independent of the experimental data (Ackermann and van den Bogert, 2010). In yielding predictive simulation for a given human motion, the determination of the cost function remains the most significant issue needed to be addressed. Generally, the cost function is represented by the combination of multiple performance criteria *via* different unknown weighting parameters (Yao et al., 2020). In such a case, determining the weighting parameters is the rite of passage to obtaining the cost function. Recent studies have suggested that determining the potential cost function for a given human motion could be cast as an inverse optimization control (IOC) problem and solved *via* the bilevel optimization technology (Mombaur et al., 2010).

Normally, a bilevel optimization problem is composed of the upper and lower levels. The optimal solution to the lower level is considered a feasible candidate for the upper level. The upper level optimizes its goals by considering its own constraints and the optimized solution obtained from the lower level (Sinha et al., 2017). Under such a case, the lower level denotes a single optimization of the movement constrained by the dynamic equation of the musculoskeletal model. The upper level adjusts the weighting parameters of the cost function to minimize the difference between the optimized solution gained from the lower level and the experimental data at each iteration (Yao et al., 2020).

Due to the nondeterministic polynomial-hard (NP-hard) nature of the bilevel optimization issue, it remains intrinsically thorny to solve this type of problems, even for simple instances (Sinha et al., 2013). To deal with this challenge, different bilevel optimization methods have been recently developed. Overall, these approaches can be categorized into classical and evolutionary methods (Sinha et al., 2018). Classical methods, such as single-level reduction using Karush–Kuhn–Tucker (KKT) conditions (Dempe et al., 2012) and gradient-based methods (Sun et al., 2020), cannot guarantee their performances over complex and large-scale bilevel optimization problems due to their lack of well-established solution procedures (Sinha et al., 2013). As alternatives to classical methods, evolutionary approaches, such as genetic algorithm (GA) (Zhang et al., 2020; Liu et al., 2022) and covariance matrix adaptation evolution strategy (He et al., 2019), have gained increasing popularity in this area, thanks to their swarm-based nature and promising abilities in handling parallel computation.

For predicting human motions *via* the bilevel optimization technology by using evolutionary algorithms, the lower level is required to generate a full predictive simulation of a given human motion based on the musculoskeletal model. Since the musculoskeletal model contains different dynamic constraints, the lower level often prefers to use the direct methods, such as the shooting method (Umberger, 2010) and the direction collocation (DC) method (Lee et al., 2016), to transfer the dynamic optimization problem into a nonlinear programming (NLP) problem in order to predict the human movement as full as possible. Compared to the shooting method, the DC method can yield a higher sparse NLP problem, which may thus enhance the computational efficacy (Lee et al., 2016). Therefore, combining with evolutionary algorithms, the DC method could be a vital alternative to the lower level optimization of the predictive simulation of human motion.

Over the last decade, applying the bilevel optimization technology to predict human motions has drawn great interest. Mombaur and Clever have used bilevel optimization to determine the underlying cost functions for human walking and running motions on different levels according to human motion capture data (Mombaur et al., 2017). A bilevel optimization method has been devised by leveraging the quadratic approximation-based method and multiple shooting method to identify potential optimality criteria of human gait generation in a constrained environment (Clever et al., 2018). Applying GA and DC, Nyuyen et al. have developed a novel bilevel optimization approach to determine the cost function in the dynamic simulation of human gait (Nguyen et al., 2019). Some other related works using bilevel optimization to determine the performance criteria of different human motions can be also found in Howard et al. (2013), Rebula et al. (2019), Price et al. (2020), Westermann et al. (2020).

From these aforementioned studies, it can be found that employing bilevel optimization to identify human motion generation is still a flourishing area. Also, based on our best knowledge, these studies mainly focus on some typical human movements, such as reaching tasks, running, jumping, or gait generation, and few of them have discussed the possibility of applying bilevel optimization to human lifting motion. Herein, this work has been devoted to investigating the potential cost function with respect to human lifting tasks *via* bilevel optimization, such that our results would provide some insights on designing and controlling the upper limbed wearable robot devices to release the physiological discomforts caused by lifting task.

To this end, blending particle swarm optimization (PSO) (Zhao et al., 2021) with the DC method, this article proposes a nested bilevel optimization approach to identify the cost function of human lifting motion. The upper level applies PSO to optimize the weighting parameter of each predefined performance criterion in the cost function minimizing the kinematic difference between the experimental data and the results gained from the lower level. The reason for using PSO can be interpreted by the fact that PSO is one of the most well-known evolutionary algorithms with excellent convergence speed, which would be suitable for dealing with our concerned issue within a tackle time (Liu, et al., 2021). Thanks to its aforementioned advantages, the DC method is implemented in the lower level to predict human kinematic and dynamic information based on the predefined cost function and human musculoskeletal model implemented into OpenSim. Finally, the performance of the proposed method is examined by experimental tests over 6 subjects, followed after a benchmark study. The experimental results confirm that the proposed method can identify a robust cost function for each subject. Thus, our method would provide practical utilities in bionics controlling of wearable robotic devices to aid human lifting motion in the future.

The rest of this article is organized as follows: Section 2 mainly presents the IOC model of human lifting motion established in this study; the proposed bilevel optimization method, including the PSO and DC method, used for identifying the cost function of human lifting motion is stated in Section 3; the experimental tests and result analysis are summarized in Section 4; and the last section completes this study by drawing conclusions and showing some future works.

## 2 Problem Statement

### 2.1 General Form of IOC Issue

It is commonly supposed that human daily motion is performed in an optimal way due to evolution, learning, and training (Mombaur et al., 2017). This, from a mathematical perspective, indicates that human motions can be formulated as optimal control problems. Yet, determining exact forms of the optimal control issues, especially as far as the establishment of the cost function, still remains a challenge due to complexities of human CNS (Lee et al., 2016). Despite optimization principles for human motions being uncertain, they can be generally denoted by the combination of multiple performance criteria (Mombaur et al., 2010). Recently, many researchers from different fields have confirmed that human motions can be mathematically formulated as an IOC model as follows (Mombaur et al., 2010, 2017; Clever et al., 2018; Nguyen et al., 2019):

$$\underset{\omega }{\mathrm{Min}}\;\;e$$ (1)

subject to

$$\omega^{lb}\leq \omega \leq \omega^{ub},$$ (2)

$$\underset{x,u}{\mathrm{Min}}J\left(\omega ,x,u,t\right),$$ (3)

$$Subject\;to:\;\dot{x}=f\left(x,u,t\right),$$ (4)

$$C^{lb}\leq C\left(x,u,t\right)\leq C^{ub},$$ (5)

where *e* indicates the distinction between the solution of the lower level and the experimental data; *ω* ∈ *R* ^*n*^ represents the unknown parameter vector in the lower level cost function for the human motion *J*, with *n* denoting the dimension of *ω*; *ω* ^*lb*^ and *ω* ^*ub*^ are the lower and upper boundaries of *ω*, respectively; *x*(*t*) ∈ *R* ^*l*^ stands for the state vector (e.g., joint positions, velocities, muscle lengths, and activations), with *l* indicating the dimension of the state; *u*(*t*) ∈ *R* ^*q*^ denotes the muscle control vector with *q* dimensions; *t* represents the execution time of a given motion; *C*_*x*,*u*,*t*_ are the boundary constraints of the lower level; and *C* ^*lb*^ and *C* ^*ub*^ are the lower and upper boundaries of *C*_*x*,*u*,*t*_.

Note that the IOC issue defined by Eqs 1–5 is a general formulation used to investigate the optimal control strategies of different human motions. Since the goals and regulation mechanisms of different motions are diversified, one needs to establish a concrete expression of the lower level cost function defined by Eq. 3 for a given human motion (Nguyen et al., 2019).

### 2.2 Modeling of Human Lifting Motion

**FIGURE 1:**
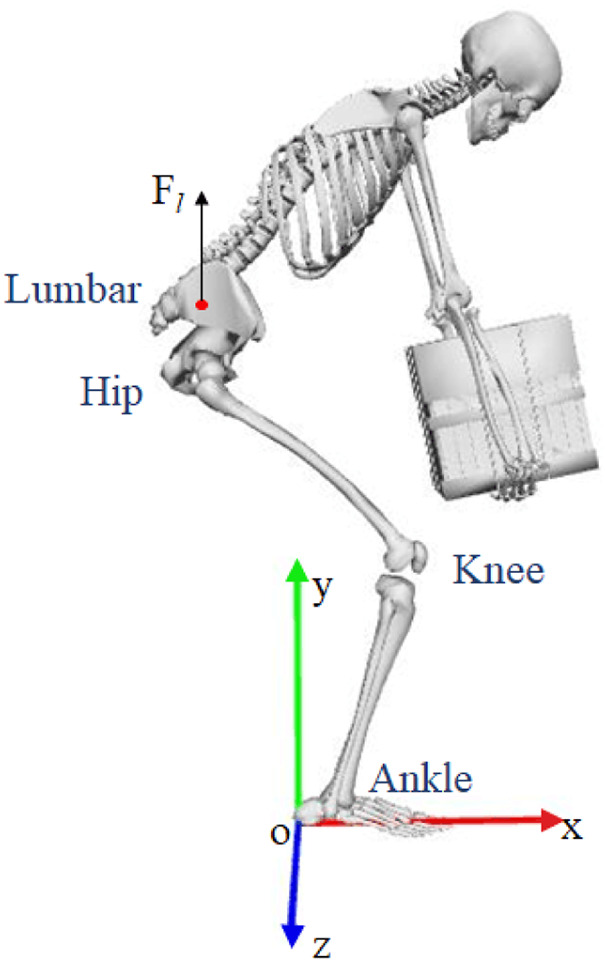
Schematic diagram of human lifting mission.

As visualized in Figure 1, this study concerns the lifting task, where a human bends down to pick up an object from the ground. In this motion, four joints, namely, the human lumbar, hip, knee, and ankle joints, are considered since they play the most significant roles in human lifting tasks. In this study, all the joints in the musculoskeletal model are fixed to flex and extension in the sagittal plane. Moreover, the joint angle is defined as negative when the joint bends in the sagittal plane. The joint angle is considered to be positive on the condition that the joint extends in the sagittal plane. Inspired by the discovery noted in the previous subsection, this study defines the upper and lower levels of this task as follows, respectively:

$$\underset{\omega }{\mathrm{Min}}\;\;e=\frac{1}{t_{f}}\int_{0}^{t_{f}}\overset{n}{\underset{j}{\sum }}{\left[x_{j}^{*}\left(t\right)-x_{j}\left(t\right)\right]}^{2}dt,$$ (6)

$$\underset{x,u}{\mathrm{Min}}J=\int_{0}^{t_{f}}\left[\omega^{\prime }\overset{n}{\underset{j}{\sum }}u_{j}^{2}\left(t\right)+\omega_{5}Com_{x}{\left(t\right)}^{2}+\omega_{6}Com_{y}{\left(t\right)}^{2}+\frac{\omega_{7}}{10^{2}}F_{l}{\left(t\right)}^{2}\right]dt,$$ (7)

where *n* denotes the number of joints (*n* = 4 in this article); *t*_*f*_ stands for the ending time of the lifting task; $x_{j}^{*}\left(t\right)$ and *x*_*j*_(*t*), respectively, indicate the predictive kinematic data from the lower level and the experimental kinematic of the *j*th joint; *j* = 1, 2, 3, 4 corresponds to human lumbar, hip, knee, and ankle, respectively; *u*_*j*_ is the joint torque of the *j*th joint; *Com*_*x*_ and *Com*_*y*_, respectively, denote positions of center mass of human body along the horizontal and vertical directions; *F*_*l*_(*t*) represents the resultant force of human lumbar in the case of bending down to pick up an object; and *ω*′ = [*ω*_1_, *ω*_2_, *ω*_3_, *ω*_4_], *ω*_5_, *ω*_6_, and *ω*_7_ are the unknown weighting parameters of different items shown in the aforementioned lower level cost function.

It is noticeable from Eq. 6 that the upper level aims to search for solutions to the kinematical information with respect to each joint from OpenSim to match the experimental kinematics as closely as possible. Since human beings always prefer to stably lift objects with least energy consumption and fatigue of lumbar, these performance metrics are considered in Eq. 7. The first item in this equation denotes the summation of total joint torque. Since joint torque can be used to calculate the energy consumption by multiplying with the angular displacement, associated with different weighting parameters, this item is used to denote the energy consumption of lifting movement. The second and third items indicate human body stability during lifting. The last item in this equation stands for the resultant force of the human lumbar. Since the human lumbar is subject to different forces, which mainly result in fatigue on the lumbar, the last item is used to evaluate fatigue in the lumbar. Moreover, due to different dimensions, this item is empirically scaled to 10^−2^ in order to obtain similar magnitudes among all items. Note that each item in Eq. 7 is calculated through the musculoskeletal model in OpenSim based on the optimized state and control variables, as well as the optimized weighting parameters. In addition, similar to some other human motions, the upper and lower levels of human lifting motion are also constrained by Eqs 2, 4, and 5, respectively.

## 3 Methods

In order to handle the bilevel optimization problem defined by Eqs 6, 7 within a tackle time, this study develops a bilevel optimization method by leveraging PSO and DC. Thanks to the promising convergence speed and parallel computation nature of PSO, this algorithm is implemented to optimize the weighting parameters by minimizing the difference between the predictive kinematics of joints obtained from the lower level and those of the experimental results. Due to the sparsity property of DC, this method is ideal for dealing with the derivative constraint, as shown in Eq. 4 (Nguyen et al., 2019). Thus, this study applies the DC method to simultaneously optimize the state and control variables in the lower level based on the optimized weighting parameters obtained from the upper level at each iteration. Note that the numerical value of each item in Eq. 7 is calculated based on the musculoskeletal model inserted into OpenSim. Applications of these two methods on the upper and lower levels are detailed in the hereinafter contents.

### 3.1 Particle Swarm Optimization

Inspired by birds flocking, Kennedy and Eberhart first proposed PSO in 1995 (Eberhart et al., 1995). Each particle in this algorithm denotes a candidate solution to an optimization issue. During the iterative search, each particle updates its position and velocity information according to its own flight experience and those of its companions as follows (Wu, et al., 2022):

$$V_{m}^{k+1}=\omega_{m}V_{m}^{k}+c_{1}r_{1}\left(pbest_{m}^{k}-X_{m}^{k}\right)+c_{2}r_{2}\left(gbest^{k}-X_{m}^{k}\right),$$ (8)

$$X_{m}^{k+1}=X_{m}^{k}+V_{m}^{k+1},$$ (9)

where $V_{m}^{k}$ and $X_{m}^{k}$ denote the velocity and position vectors of the *m*th particle at iteration *k*, respectively; *ω*_*m*_ is a real coefficient, standing for the inertia weight parameter of particle *m*; *c*_1_ and *c*_2_ are two positive real parameters, respectively, denoting the cognitive and social acceleration parameters; *r*_1_ and *r*_2_ are two random numbers uniformly distributed in [0, 1]; and $pbest_{m}^{k}$ and *gbest* ^*k*^ represent the personal best position of the *m*th particle and the global best position of the swarm at iteration *k*, respectively.

In the case of applying PSO to solve the upper level optimization problem of human lifting motion, the position vector of each particle is encoded by the unknown weighting parameters. Since 7 weighting parameters are considered in this study, the length of the position vector of each particle equals 7. During the search process, the fitness value of each particle is calculated based on Eq. 6. It is notable that the integral operator in Eq. 6 is approximately handled by the summation of *N*_*t*_ discretized time nodes between 0 and *t*_*f*_ in terms of calculating the fitness value of each particle (*N*_*t*_ = 101 in this article).

During the search process, in order to guarantee the searched weighting parameters to satisfy the constraint given by Eq. 2, each dimension of the position vector of each particle is modified by the following saturation strategy:

$$\omega_{i}=\left\{\begin{array}{c} \omega_{i}^{ub},\;if\;\omega_{i}>\omega_{i}^{ub}\;\\ \omega_{i}^{lb},\;if\;\omega_{i}<\omega_{i}^{lb}\;\\ \omega_{i},\;otherwise\; \end{array}\right.,$$ (10)

where $\omega_{i}^{lb}$ and $\omega_{i}^{ub}$ are the lower and upper boundaries of the *i*th weighting parameter *ω*_*i*_, respectively.

The algorithmic steps of applying PSO to search the optimized weighting parameters in the upper level are shown in Table 1. *k*_max_ and *N*_*p*_, respectively, denote the maximum iteration number and swarm size in this table. The evolution of PSO will not exist until the iteration number reaches *k*_max_. It is notable from this table that the predictive kinematic data are obtained from the lower level in terms of calculating the fitness value of each particle based on Eq. 6.

**TABLE 1:** Algorithmic steps of using PSO to solve the upper level of human lifting motion.

1	Set the needed simulation parameters and randomly generate an initialize swarm
2	**while** *k* ≤ *k*_max_ **do**
3	**for** *m* = 1: *N*_*p*_ **do**
4	Calculate the fitness value of particle *m* based on Eq. 6
5	Update the velocity information of particle *m* based on Eq. 8
6	Update the position information of particle *m* based on Eq. 9
7	Modify the position vector of particle *m* based on the saturation strategy given by Eq. 10
8	Update $pbest_{m}^{k}$ of particle *m*
9	**end for**
10	Update the global best solution *gbest* ^*k*^ of the swarm and send *gbest* ^*k*^ to the lower level optimizer
11	Increase the iteration number *k* by 1
12	**end while**
13	Output $gbest^{k_{\max}}$ as the optimized weighting parameters

### 3.2 Direct Collocation Method

The DC method has been used to transfer the lower level issue of lifting motion into an NLP problem in virtue of its sparse nature. To this end, the state and control variables in Eq. 7 are first discretized along the time axis as follows:

$$z=\left(x_{1},x_{2},\ldots ,x_{N},u_{1},u_{2},\ldots ,u_{N},t_{f}\right),$$ (11)

where *N* stands for the number of nodes and *t*_*f*_ is the time final. Notice that, according to our pilot test, *N* is empirically set to be 25 in this article so as to release the burden of the computation time of the lower level.

The dynamic constraint of the lower level given by Eq. 4 can be then simplified into an equation constraint through the mid-point method as follows:

$$x_{j+1}-x_{j}=\frac{1}{2}\left(t_{j+1}-t_{j}\right)\left[f\left(x_{j+1},u_{j+1},t_{j+1}\right)+f\left(x_{j},u_{j},t_{j}\right)\right],$$ (12)

where *x*_*j*+1_ and *x*_*j*_ are values of the (*j* + 1)^*th*^ and *j*th discretized state variables, respectively; (*t*_*j*+1_ − *t*_*j*_) is the time difference between the (*j* + 1)^*th*^ and *j*th nodes; *u*_*j*+1_ and *u*_*j*_ are values of the (*j* + 1)^*th*^ and *j*th discretized control variables, respectively; and *f* (*x*_*j*+1_, *u*_*j*+1_, *t*_*j*+1_) and *f* (*x*_*j*_, *u*_*j*_, *t*_*j*_) are values of the dynamic constraints at (*j* + 1)^*th*^ and *j*th nodes, respectively.

Following the way noted previously, the lower level issue is transferred into an NLP problem, which can be solved by a typical NLP solver. In this study, the SNOPT solver (Andrei, 2017) is used to handle the lower level NLP issue due to its simplicity. At each iteration, the lower level implements SNOPT to optimize the defined objective function given by Eq. 7 based on the obtained weighting parameters in the upper level. Note that the integral operation in this objective function can be instituted by summation calculation since the state and control variables are discretized along the time axis. Moreover, in the case of applying SNOPT to solve the lower level problem, the boundary constraints of the state and control variables, as shown in Eq. 5, can be similarly modified by Eq. 10 by setting appropriate values of the lower and upper boundaries. The reader can refer to Andrei (2017) for more detailed information on using SNOPT to solve different NLP issues.

## 4 Experimental Test and Result Analysis

Followed after a simple benchmark study, the developed bilevel optimization method is then verified by experimental tests on six subjects. The obtained results and analysis of the benchmark study and experimental tests are stated in the following contents.

### 4.1 Benchmark Study

In the conducted benchmark study, an IOC problem with a known optimal solution is used in this study as follows (Mcasey et al., 2012):

$$\underset{\omega }{\mathrm{Min}}\;\;e={\left(x\left(t\right)-0.28197\right)}^{2}$$ (13)

subject to

0≤ω≤20, (14)

$$\underset{x,u}{\mathrm{Min}}J=\int_{0}^{1}\left(x^{2}\left(t\right)+\omega u^{2}\left(t\right)\right)dt,$$ (15)

$$Subject\;to:\;\dot{x\left(t\right)}=-x\left(t\right)+u\left(t\right),$$ (16)

$$x\left(0\right)=1.$$ (17)

**FIGURE 2:**
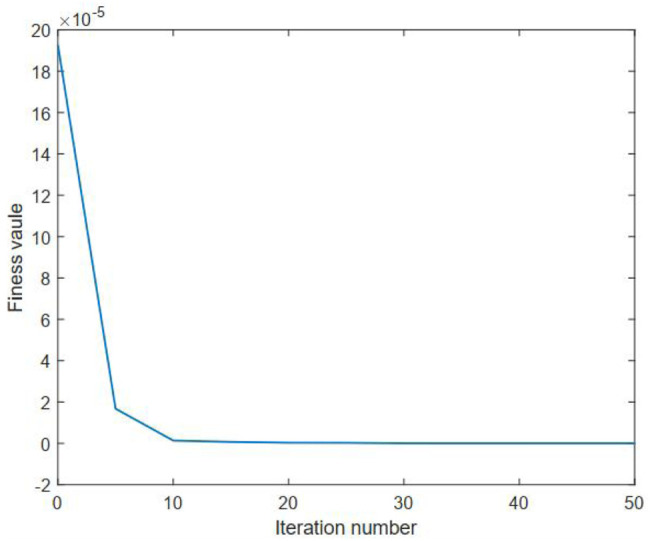
Average fitness curve of the upper level for the benchmark test function.

**FIGURE 3:**
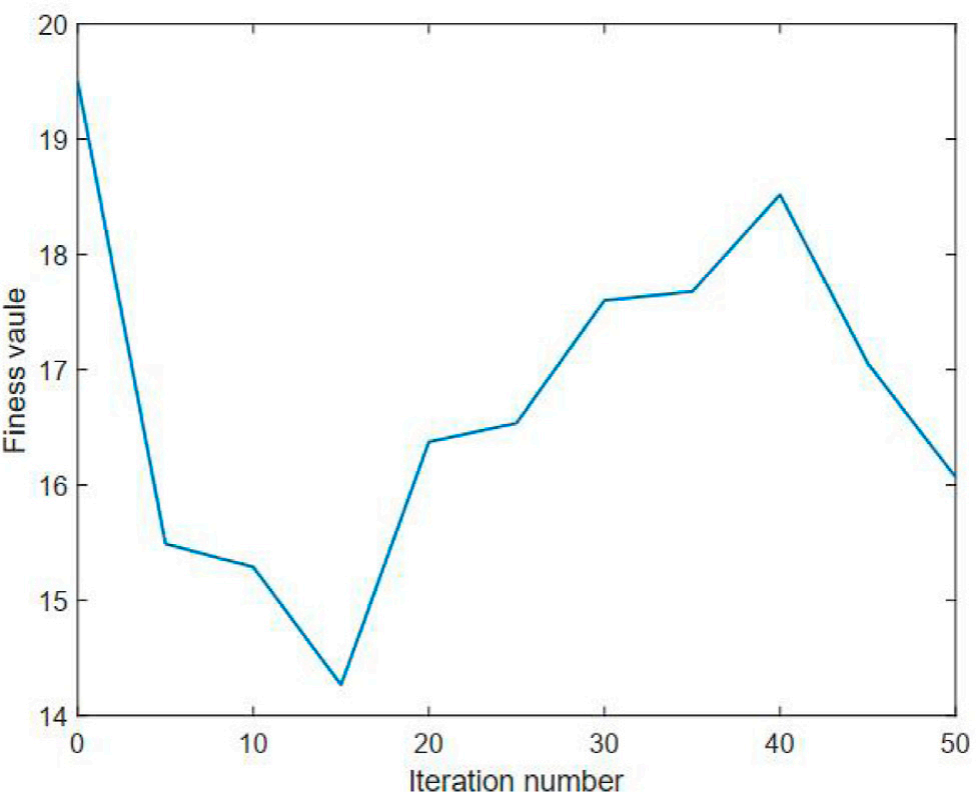
Average fitness curve of the lower level for the benchmark test function.

The aforementioned benchmark test function has an optimal solution with ω* = 1. In our benchmark study, a Monte Carlo test with 10 runs is conducted in order to reduce the random effects. The needed simulation parameters of PSO are given in Table 2. The number of nodes of the DC method is empirically set to be 25. The statistical results of using the developed method on the benchmark test function are summarized in Table 3. The average fitness value curves of the upper and lower levels for solving this benchmark are visualized in Figures 2, 3, respectively.

**TABLE 2:** Simulation parameters of PSO in the upper level for the benchmark study.

Parameter item	Parameter value
*ω*_*m*_	0.9
*c*_1_	2
*c*_2_	2
*N*_*p*_	40
*k*_max_	50

**TABLE 3:** Statistical results gained by the proposed method for the benchmark test function (“NS” represents the numerical solution of *ω*.“ULFV” and “LLFV” denote the fitness values of the upper and lower levels, respectively. “CT” indicates the computation time.“Std.” denotes the standard deviation.).

	NS (*ω*)	ULFV	LLFV	CT (s)
Best	0.9530	1.2179E-08	11.02	1.18E+02
Worst	0.9454	2.2133E-07	19.86	1.22E+02
Average	0.9473	2.4263E-08	16.06	1.19E+02
Std.	0.0019	6.5787E-08	5.847	1.01E+00

It can be observed from Table 3 that the proposed method can provide an average numerical solution of 0.9473 for the benchmark test function, with a standard deviation of 1.90E-03. It can be also observed from this table that the mean cost function values of the upper and lower levels gained by using the proposed method for the benchmark are 2.4263E-08 and 16.06, respectively. Also, one can note from this table that the proposed method averagely takes around 1.19E+02 s to cope with the selected benchmark test function. Thus, these observations, to some extent, can reflect the effectiveness of our proposed method on the bilevel optimization issue. Moreover, it is clear from Figures 2, 3 that the average fitness value curve of the upper level keeps decreasing with the iteration number increasing, whereas the average fitness value curve of the lower level oscillates. This observation is due to the fact that the bilevel optimization issue is naturally hierarchical (Sinha et al., 2018). The optimal solution to the lower level is a candidate, rather than the optimal, solution to the upper level. Thus, the optimality of the upper level always has priority over that of the lower level.

### 4.2 Experimental Studies on Different Subjects

To verify the developed method on the IOC issue of human lifting motion, six healthy male subjects (age: 23 ± 2 years, mass: 62.5 ± 4.52 kg, height: 1.71 ± 0.101 m) have been recruited to carry out a pack with 10 kg in our experimental study. The experimental kinematic data of each subject is obtained by using a motion capture system (Nokov, Beijing Metrology Technology Co., Ltd.) with 39 marker points. For more details about ways of sticking markers, the reader can refer to the operation guidance from OpenSim official website (https://simtk-confluence.stanford.edu:8443/display/OpenSim/Gait+2392+and+2354+Models). After gaining experimental kinematic data, the musculoskeletal model is scaled by the scaling tool in OpenSim to change the anthropometry of the model in order to match the specific subject as far as possible. Note that joint kinematics and kinetics data (e.g., joint angles and torques) are gained by inverse kinematics and inverse dynamics tools in OpenSim.

**FIGURE 4:**
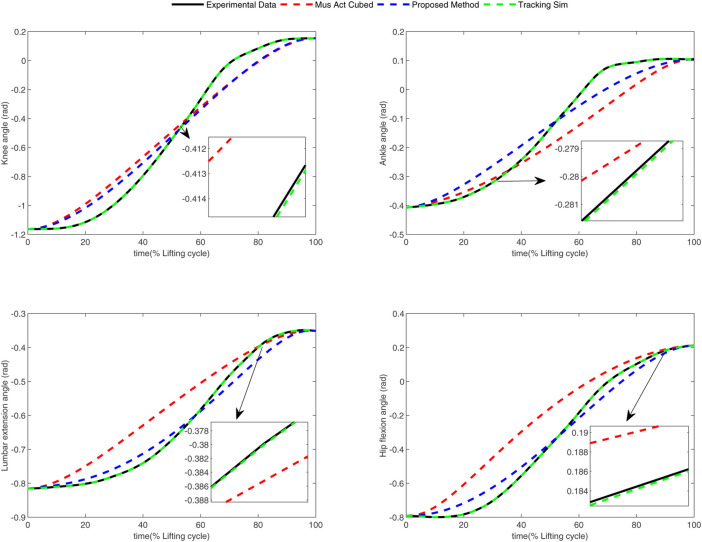
Kinematics results of different joints obtained by different methods for subject 1.

**FIGURE 5:**
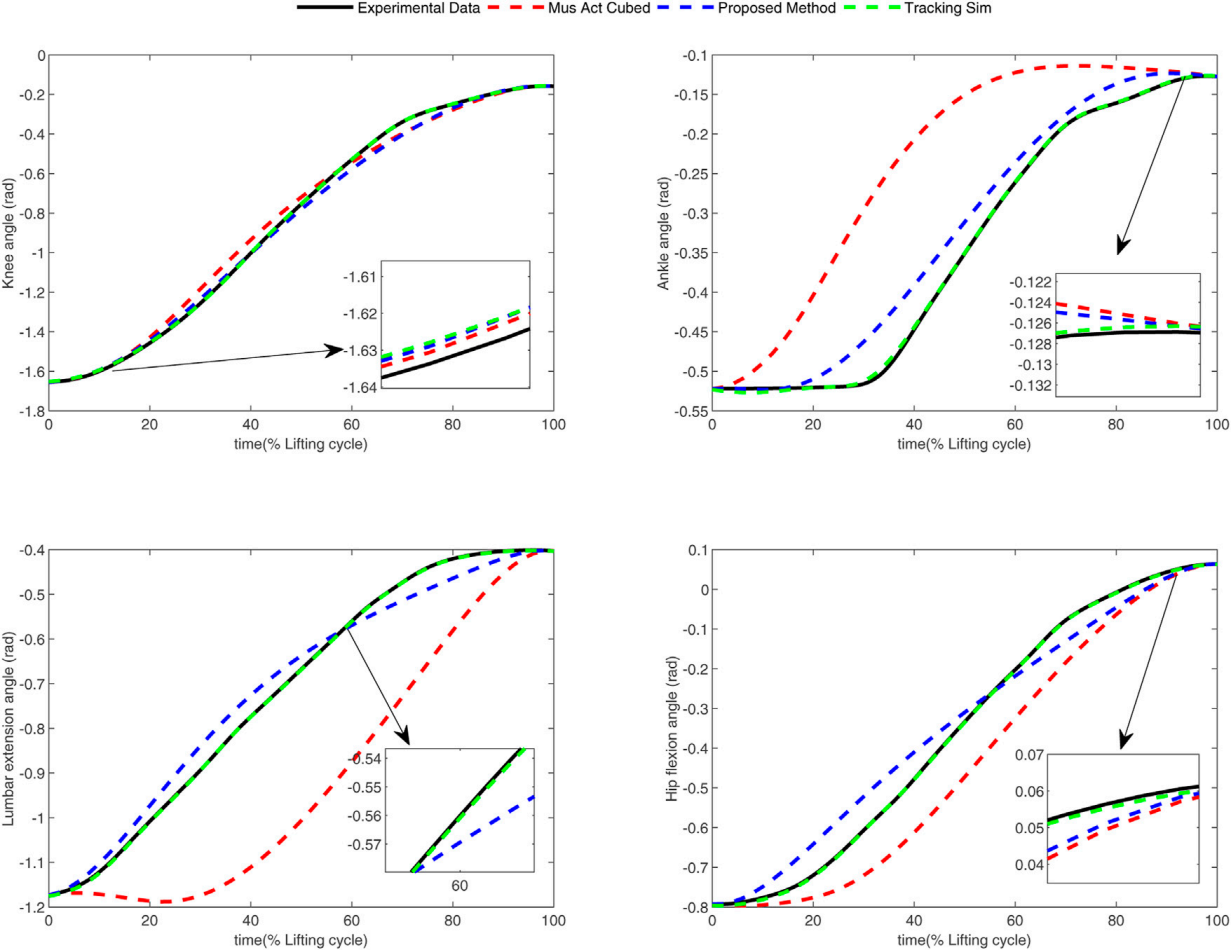
Kinematics results of different joints obtained by different methods for subject 2.

**FIGURE 6:**
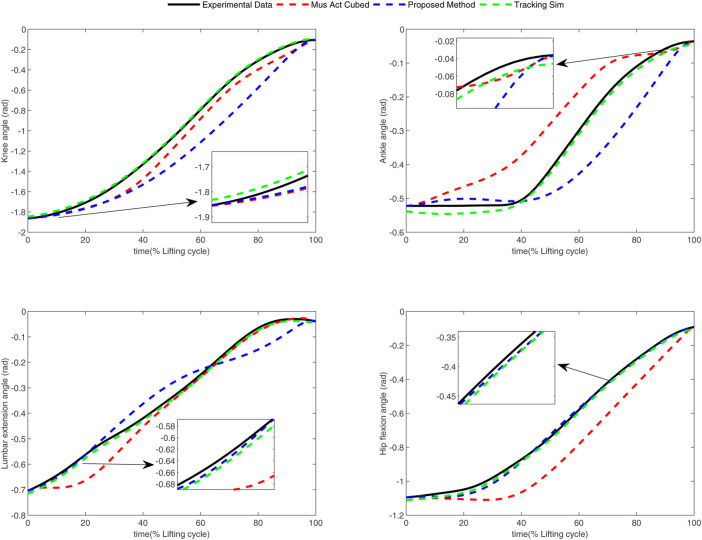
Kinematics results of different joints obtained by different methods for subject 3.

**FIGURE 7:**
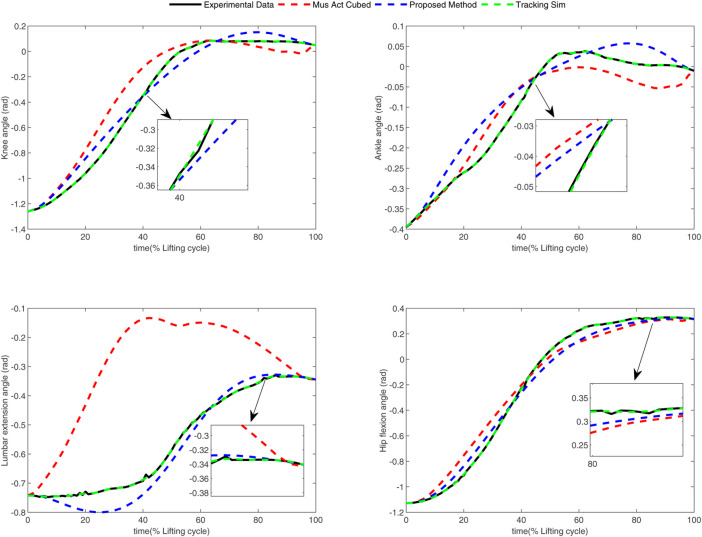
Kinematics results of different joints obtained by different methods for subject 4.

**FIGURE 8:**
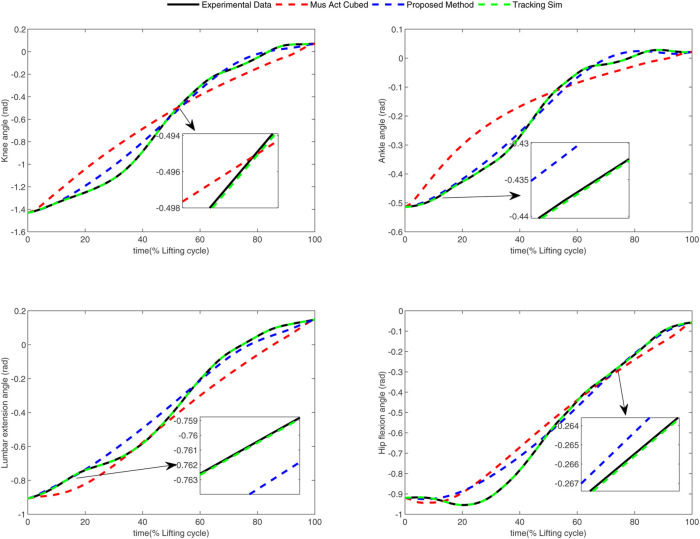
Kinematics results of different joints obtained by different methods for subject 5.

**FIGURE 9:**
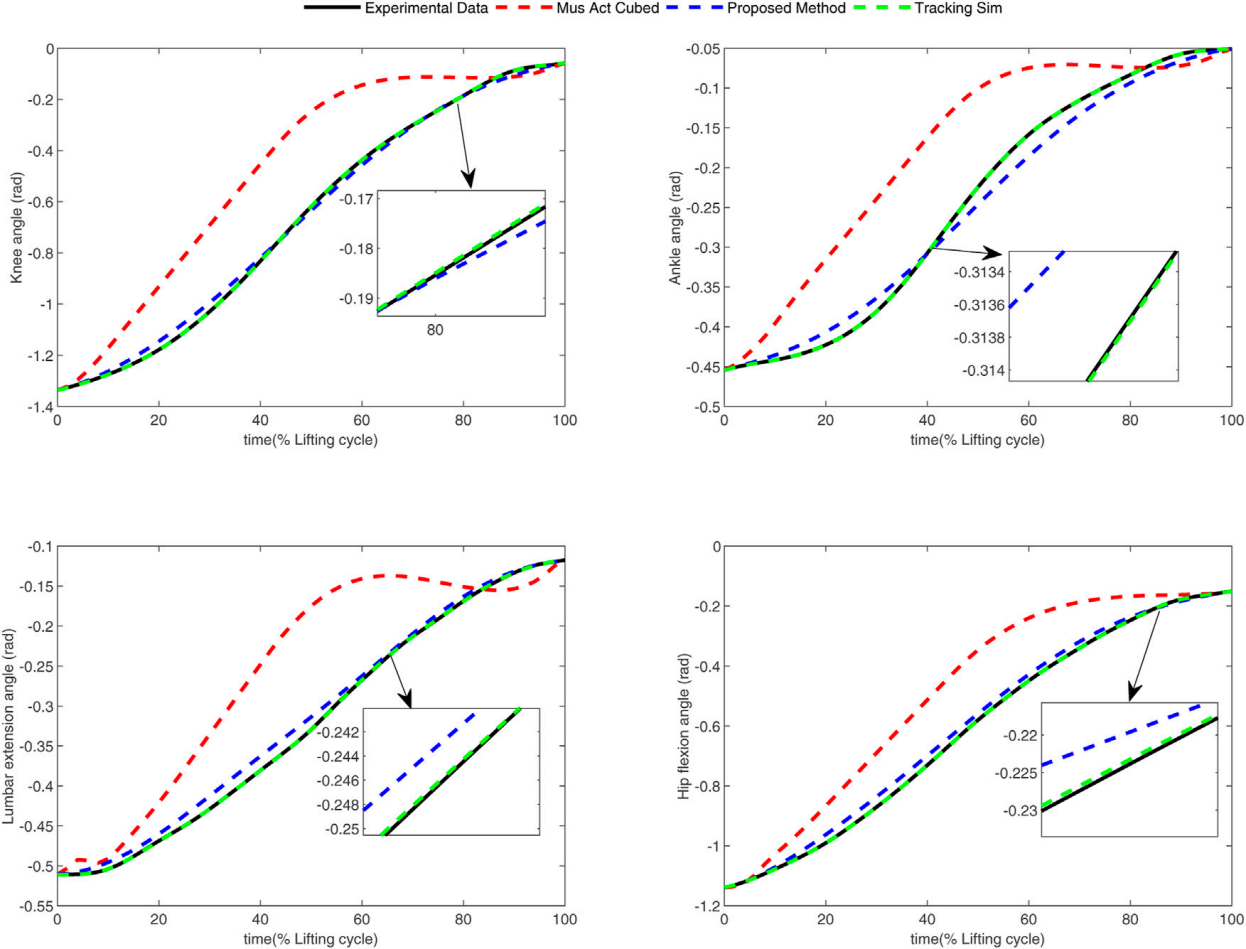
Kinematics results of different joints obtained by different methods for subject 6.

**FIGURE 10:**
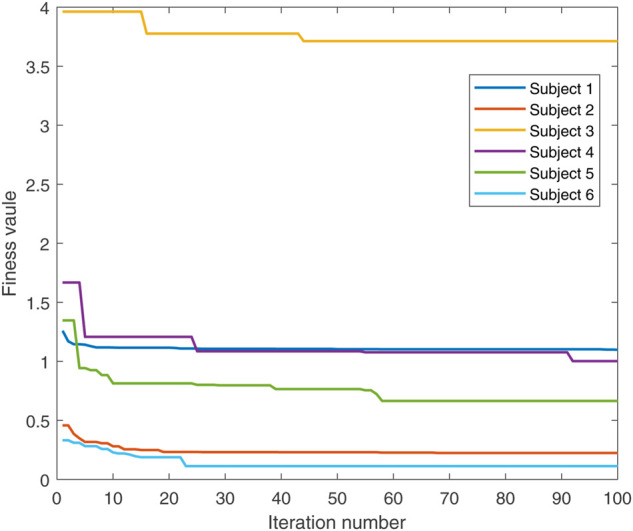
Upper fitness value curves searched by the proposed method for the six subjects.

In the conducted experimental tests, each weighting parameter is set to locate within [0,1] for each subject. The maximum number of iterations of PSO is set to be 100. The rest simulation parameters of PSO and DC are referred to Section 4.1. The final experimental results of each subject obtained by using the developed bilevel optimization method are compared with those gained by the tracking simulation method (Koelewijn et al., 2016) (referred to as “Tracking Sim” in this article ) and the muscle activation cubed approach (Nguyen et al., 2019) (referred to “Mus Act Cubed” in this study). Note that only the energy performance metric is considered in the lower level cost function in “Mus Act Cubed,” for instance, only the first four items shown in Eq. 7 are considered in the “Mus Act Cubed” method. The kinematics results of different joints obtained by using the different methods for different subjects are illustrated in Figures 4–9, respectively. The numerical results of each considered joint and optimized weighting parameters obtained by different methods for each subject are reported in Table 4, where the best result of each item is highlighted in boldface. The upper fitness value curves searched by using our proposed method for the six subjects are illustrated in Figure 10.

**TABLE 4:** Numerical results of each considered joint and optimized weighting parameters of different methods for different subjects (where “NAN” means “unavailable” and “ULFV” indicates “upper level fitness value”).

Subject	Method	Optimized weighting parameters	Joint error				ULFV
			Lumbar	Hip	Knee	Ankle	
1	Tracking	NAN	**2.74E-08**	**5.30E-08**	**5.38E-08**	**2.36E-08**	**1.11E-04**
	Mus Act	[0.937, 0.066, 0.852, 0.005]	5.80E-03	3.41E-02	1.35E-02	5.40E-03	4.10E-00
	Proposed	[0.248, 0.317, 0.567, 0.778, 0.534, 0.975, 0.778]	5.00E-04	2.90E-03	9.70E-03	2.20E-03	1.09E-00
2	Tracking	NAN	**2.53E-06**	**6.37E-06**	**7.75E-06**	**9.14E-06**	**1.60E-03**
	Mus Act	[0.250, 0.036, 0.740, 0.405]	8.13E-02	1.26E-02	2.40E-03	2.86E-02	5.32E-01
	Proposed	[0.536, 0.388, 0.354, 0.075, 0.915, 0.919, 0.237]	1.70E-03	3.60E-03	1.20E-03	1.40E-03	2.23E-01
3	Tracking	NAN	**1.00E-04**	**2.18E-04**	**2.61E-04**	**2.61E-04**	**7.81E-02**
	Mus Act	[0.676, 0.135, 0.035, 0.600]	2.00E-03	2.07E-02	7.90E-03	7.00E-03	5.17E-00
	Proposed	[0.094, 0.324, 0.187, 0.473, 0.374, 0.791, 0.761]	1.90E-03	3.00E-04	4.40E-02	6.30E-03	3.71E-00
4	Tracking	NAN	**7.86E-06**	**1.31E-05**	**2.10E-06**	**7.40E-07**	**1.10E-03**
	Mus Act	[0.159, 0.028, 0.693, 0.749]	2.12E-01	1.32E-02	3.69E-02	3.50E-03	1.37E-00
	Proposed	[0.679, 0.744, 0.622, 0.326, 0.700, 0.340, 0.274]	2.90E-03	5.30E-03	5.30E-03	2.80E-03	1.00E-00
5	Tracking	NAN	**7.27E-08**	**1.21E-07**	**6.53E-08**	**4.36E-08**	**1.78E-04**
	Mus Act	[0.289, 0.328, 0.701, 0.380]	9.00E-03	6.30E-03	3.43E-02	9.90E-03	3.51E-00
	Proposed	[0.427, 0.671, 0.264, 0.932, 0.741, 0.449, 0.772]	2.90E-03	3.40E-03	4.50E-03	3.00E-04	6.64E-01
6	Tracking	NAN	**5.78E-08**	**8.33E-08**	**6.69E-08**	**9.47E-09**	**1.91E-04**
	Mus Act	[0.707, 0.872, 0.392, 0.447]	8.50E-03	2.57E-02	6.76E-02	9.00E-03	5.17E-01
	Proposed	[0.848, 0.397, 0.230, 0.685, 0.392, 0.533, 0.153]	1.00E-04	5.00E-04	4.01E-04	2.00E-04	1.12E-02

The best simulation results among different methods.

It is evident from Figures 4–9 that the kinematics of each joint optimized by the three methods can almost follow those of the corresponding reference data. This can reflect the effectiveness of each method for solving the predictive simulation problem of human lifting motion. It can be found from Figure 10 that the upper level fitness value curves searched by using the proposed method for the six subjects keep decreasing in the early phase of the evolution. This would imply the efficacy of standard PSO in the upper level optimizer in our developed method. Here, we need to mention that the fitness value curves searched by standard PSO may probably fall into local optimums for the 6 subjects since the fitness value curves remain stable in the latter of the evolution, as demonstrated in Figure 10. This observation is more likely due to the fact that standard PSO cannot keep a good balance between its global and local search powers. Thus, we are considering the possibility of developing some updating strategies to well balance such two capabilities of PSO in our upcoming study so that the performance of the developed method could be further enhanced.

It can be also found from Table 4 that the “Tracking Sim” method is followed by our proposed method and the “Mus Act Cubed” method in terms of the joint error and optimization fitness value. It is important to note from Table 4 that despite providing the best tracking performance among the three considered methods, the “Tracking Sim” method cannot obtain the determined weighting parameters. This indicates that the “Tracking Sim” method cannot gain an analytical expression of the optimal control strategy of human lifting motion. The reason that the “Tracking Sim” method cannot analytically solve the predictive simulation problem of human lifting motion can be explained by the fact that this method is merely a data-driving optimization method.

Moreover, one can claim from Table 4 that our proposed method and the “Mus Act Cubed” method can analytically determine the optimal control strategy of human lifting motions since these two approaches can obtain the weighting parameters. Due to the distinctions of human physiological structures such as different weights and heights, the weighting parameters shown in Table 4 for each subject searched by using these two approaches are diversified. Compared to the “Mus Act Cubed” method, the proposed method can generate a more accurate analytical expression of the optimal control strategy involved in human lifting motions. This is because those three more optimization items are considered in the lower level cost function in our proposed method. This means that combining multiple performance criteria in the lower level cost function results in a more realistic simulation of the human lifting motion model, which however leads to the determination of the motion model to be more computationally expensive. This discovery can be supported by the truth that the proposed method averagely takes about 74 h (with around 20 h for the lower level optimizer) to complete the programming run for each subject, which generally needs more than 12 h than those of the “Mus Act Cubed” method. However, since the predictive simulation of human lifting motion is conducted off-line, the computation time would not be the most important factor in the determination of the cost function of human lifting motion. Based on the aforementioned analysis, it may allow us to conclude that our proposed method could be considered as a vital alternative in the field of the predictive simulation of human lifting motion.

## 5 Conclusion and Future Works

Aiming at investigating the underlying cost function of human lifting missions, this study develops a nested bilevel optimization approach based on PSO and DC methods. In the developed method, the upper level implements PSO to optimize the weighting parameters among different performance criteria with the goal of minimizing the kinematic error between the experimental data and the result predicted from the lower level optimizer. The lower level optimizer applies the DC method to predict human kinematic and dynamic information based on the human musculoskeletal model inserted into OpenSim. The efficiency of the developed method is verified by a benchmark study as well as the experimental tests over six subjects. The simulation results confirm that our proposed method is capable of finding the cost function of human lifting motion within a tackling time. Therefore, our method could be regarded as a paramount alternative in the prediction simulation of human motion and would benefit the bionic control of different wearable devices.

The method and results shown in this article arise several issues that deserved some future studies. Although none of the weighting parameters obtained by using the proposed method are close to zero, the values of the weighting parameters are diversified over different subjects. Thus, developing an approach to evaluate the relative importance of a performance criterion deserves further strives. Since the global and local search capabilities of PSO heavily affect its optimization performance and convergence speed, developing more advanced updating strategies by mixing PSO with some other approaches such as Faster RCNN (Jiang, et al.., 2021) or rumor diffusion process (Chen et al., 2022) to enhance such two abilities of PSO could be the second significant issue in the near future to further improve and optimize the performance and the computation time of the upper level optimizer. Moreover, we are considering the possibility of comparing the developed approach with some other evolutionary algorithms by adding several more performance criteria in the lower level cost function in our shortcoming studies. Last but not least, we are striving to use the proposed method for the bionic control of upper limbed wearable robot devices to assist human lifting motion. For this potential application, the proposed method could be first applied to identify the personalized optimal control law for each subject. The determined optimal control strategy can then be mapped into the control system of a wearable robotic device, such that the device can achieve human-like control behavior during assisting human lifting motion.

## Data Availability

The original contributions presented in the study are included in the article/Supplementary Material, further inquiries can be directed to the corresponding author.
